# Baseline autoantibody profile in rheumatoid arthritis is associated with early treatment response but not long-term outcomes

**DOI:** 10.1186/s13075-018-1520-4

**Published:** 2018-02-26

**Authors:** Emma C. de Moel, Veerle F. A. M. Derksen, Gerrie Stoeken, Leendert A. Trouw, Holger Bang, Robbert J. Goekoop, Irene Speyer, Tom W. J. Huizinga, Cornelia F. Allaart, René E. M. Toes, Diane van der Woude

**Affiliations:** 1 Leiden University Medical Center, Leiden, The Netherlands; 2 Orgentec Diagnostika GmbH, Mainz, Germany; 3 Haga Hospital, the Hague, The Netherlands; 4 Haaglanden Medical Center, the Hague, The Netherlands

**Keywords:** Ant-CCP, Rheumatoid arthritis, Rheumatoid factor, Autoantibodies, Disease activity

## Abstract

**Background:**

The autoantibody profile of seropositive rheumatoid arthritis (RA) is very diverse and consists of various isotypes and antibodies to multiple post-translational modifications. It is yet unknown whether this varying breadth of the autoantibody profile is associated with treatment outcomes. Therefore, we investigated whether the composition of the autoantibody profile in RA, as a marker of the underlying immunopathology, influences initial and long-term treatment outcomes.

**Methods:**

In serum from 399 seropositive patients with RA in the IMPROVED study, drawn at baseline and at the moment of drug tapering, we measured IgG, IgM, and IgA isotypes for anti-cyclic citrullinated peptide-2 and anti‐carbamylated protein antibodies, IgM and IgA rheumatoid factor, and reactivity against four citrullinated and two acetylated peptides (anti-modified protein antibodies (AMPAs)). We investigated the effect of the breadth of the autoantibody profile on (1) change in disease activity score (DAS)44 between 0 and 4 months, (2) initial drug-free remission (DFR, drug-free DAS44 < 1.6) achieved between 1 and 2 years of follow up, and (3) long-term sustained DFR until last follow up.

**Results:**

Patients with a broad autoantibody profile at baseline had a significantly better early treatment response: ΔDAS 0–4 months of 1–2, 3–4, and 5–6 vs 7–8 isotypes, -1.5 (*p* < 0.001), -1.7 (*p* = 0.03), and -1.8 (*p* = 0.04) vs -2.2. Similar results were observed for AMPA number. However, patients with a broad baseline autoantibody profile achieved less initial DFR. For long-term sustained DFR there was no longer an association with the breadth of the autoantibody response. When assessing autoantibodies at the moment of tapering, similar trends were observed.

**Conclusions:**

A broad baseline autoantibody profile is associated with a better early treatment response. The breadth of the baseline autoantibody profile, reflecting a break in tolerance against several different autoantigens and extensive isotype switching, may indicate a more active humoral autoimmunity, which could make the underlying disease processes initially more suppressible by medication. The lack of association with long-term sustained DFR suggests that the relevance of the baseline autoantibody profile diminishes over time.

**Trial registration:**

ISRCTN11916566. Registered on 7 November 2006. EudraCT, 2006- 06186-16. Registered on 16 July 2007.

**Electronic supplementary material:**

The online version of this article (10.1186/s13075-018-1520-4) contains supplementary material, which is available to authorized users.

## Background

Patients with rheumatoid arthritis (RA), a chronic autoimmune disease primarily affecting the joints, harbour autoantibodies recognizing several post-translationally modified peptides. The most well-characterised of these are anti-citrullinated peptide 2 (anti-CCP2) antibodies and rheumatoid factor (RF) that are present in approximately 60% of patients. Anti-CCP2 and RF-positive patients have a worse long-term prognosis and are less likely to achieve drug-free remission (DFR) [1–5]. Whether they also differ in early treatment response is controversial [5–8].

However, considering only these two autoantibodies may be oversimplifying a complex picture. Novel RA-associated autoantibody systems such as anti-carbamylated protein (anti-CarP) [9, 10] and anti-acetylated protein antibodies [11] continue to be identified. Moreover, the autoantibody profile is very diverse, with antibodies targeting variable numbers of different peptides with the same post-translational modification, and with marked heterogeneity in isotype usage [12–14]. This diversity in the breadth of the autoantibody profile most likely reflects the break of tolerance to multiple autoantigens and the maturity of the humoral autoimmune response [15–17].

It is currently unknown to what extent the breadth of the autoantibody profile influences treatment outcomes. In RA, early initiation of disease-modifying anti-rheumatic drugs (DMARDs) and treat-to-target strategies have improved clinical remission rates [18, 19] and in some patients tapering and withdrawal of DMARDs can be attempted, but not all patients successfully become symptom-free or drug-free. There is a growing need to understand the mechanisms that set apart patients that do achieve early clinical remission or long-term sustained DFR (the closest approximation of disease curative available) [3, 20, 21].

Since autoantibodies are linked to both RA pathophysiology and treatment outcomes, they offer a unique perspective to shed light on the pathophysiological mechanisms underlying RA chronicity. Given the varying composition of the RA autoantibody profile (with its diversity in autoantigen recognition and extensive isotype switching), it appears plausible that the breadth of this profile could be associated with treatment outcomes. No studies to date have investigated the effect of composition of the baseline autoantibody profile on early response to conventional DMARD therapy or long-term DFR. Furthermore, it is also unclear whether the breadth of the profile present at baseline or at the moment of drug-tapering (or both) is more indicative the ability of a patient to reach and maintain DFR. To fill these niches in knowledge, we investigated whether outcomes such as early treatment response to DMARDs and DFR are associated with the breadth of the autoantibody profile at baseline in seropositive patients with RA and at the moment of drug-tapering.

## Methods

### Study design

The Induction therapy with Methotrexate and Prednisone in Rheumatoid Or Very Early arthritic Disease (IMPROVED) study is a multicentre, randomized controlled trial that enrolled 610 patients with early (< 2 years) untreated RA or undifferentiated arthritis. It was aimed at change in the disease activity score-remission (DAS44 < 1.6), and for those achieving remission, aimed at drug-free remission (DFR), with treatment adjusted every 4 months according to whether treatment targets had been reached. Initial treatment comprised methotrexate (MTX) and high-dose prednisone, followed by either tapering of medication or randomization to one of two treatment arms: MTX, prednisone, hydroxychloroquine, and sulphasalazine combination (multi-DMARD arm) or MTX and adalimumab combination as described previously [2]. According to the protocol, patients tapered and discontinued methotrexate at 8 months if they achieved early remission, allowing them to become drug-free and remain so until the DAS increased to > 1.6.

### Patient selection and outcomes

**Fig. 1:**
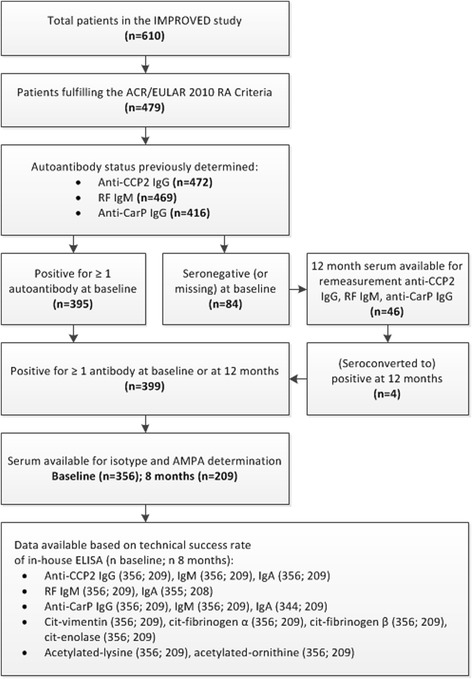
Extended patient selection algorithm. ACR, American College of Rheumatology; EULAR, European League Against Rheumatism; RA, rheumatoid arthritis; anti-CCP2, anti-citrullinated protein 2; RF, rheumatoid factor; anti-CarP, anti-carbamylated protein antibodies; AMPA, anti-modified protein antibodies; Cit, citrullinated

All 479 patients fulfilling the 2010 American College of Rheumatology (ACR)/European League Against Rheumatism (EULAR) RA criteria were selected. Of these patients, those seropositive at baseline by routine clinical testing for anti-CCP2 IgG or RF IgM, or by our in-house assay for anti‐CarP IgG [10], were selected (*n* = 395; see Fig. 1 for detailed selection algorithm). If patients were fully seronegative at baseline, we measured anti-CCP2 IgG, RF IgM, and anti-CarP IgG in serum collected after 1 year of follow up to include any patients that seroconverted to positive, yielding 399 seropositive patients, of whom 356 had baseline, untreated serum available and 209 had 8-month treated serum available for further serological measurements as described subsequently [2].

Main outcomes we investigated were initial DAS change from baseline to 4 months (ΔDAS 0–4 months) and DFR. DAS change from baseline to 4 months occurred under treatment with MTX and high-dose prednisone. DFR was defined as the ability to discontinue medication and remain in remission for (at least) 1 year after achieving DAS44 < 1.6. We differentiated between initial DFR and long-term sustained DFR. Initial DFR was defined as DFR between 1 and 2 years of the study, which due to the protocol could only be achieved by patients that were in early DAS remission at 4 months after tapering prednisone and MTX. Long-term sustained DFR was defined as DFR of at least 1 year duration until the last follow up in all patients (including those who were randomized to the multi-DMARD or adalimumab treatment arm), which is the closest approximation of disease cure currently available for RA. Due to the protocol design, the group of patients that could achieve initial DFR was smaller than (i.e. a subgroup of) all patients who could achieve long-term sustained DFR.

### Serological measurements

Enzyme-linked immunosorbent assay (ELISA) was used essentially as described previously to measure anti-CCP2 IgG, IgM, and IgA [22], RF IgM and IgA [12], and anti-CarP IgG, IgM, and IgA [10, 23] in baseline serum from untreated patients. We also conducted fine-specificity ELISA for IgG directed against four citrullinated peptides: citrullinated-vimentin 59-74, citrullinated-fibrinogen β 36-52 and α 27-43, and citrullinated-enolase 5-20 [24]. Last, ELISA for anti-acetylated lysine and anti-acetylated ornithine IgG (Orgentec Diagnostika GmbH, Germany) was performed as previously described [11].

Absorbance was converted to arbitrary units per millilitre (aU/mL) using a standard curve of pooled, serially diluted highly positive patient serum. Samples were considered positive if they fell above the cutoff of the mean aU/mL value plus two standard deviations in serum samples from 76 healthy controls from the Leiden area, run in tandem with the samples for each ELISA. Because antibodies may be aspecifically directed to the unmodified variant of the peptide/protein of interest, we applied a specificity criterion to each ELISA. For anti-CCP2 IgA and IgM, the difference between the citrullinated and unmodified optical density (OD) had to be more than 0.1; for anti-CarP and anti-acetylated peptide ELISAs, the difference (aU/mL) between the modified and unmodified signal had to be above the cut-off. Since previous experiments revealed minimal aspecific signals for the citrullinated fine-specificity ELISA, no specificity criterion was applied.

The technical success rate of the ELISA was at least 96% (Fig. 1). There was good agreement in positivity between the original baseline measurement performed routinely for anti-CCP-antibodies and RF at inclusion and the in-house baseline re-measurement (Additional file 1: Figure S1).The first and second in-house measurement of anti-CarP IgG showed fair agreement (Additional file 1: Figure S1). Positivity for the various isotypes measured largely overlapped (Additional file 1: Figure S2).

### Statistical analysis

We constructed categories reflecting the breadth of the antibody response that consisted of the sum of positive antibody tests. First, the number of isotypes present; both in total (anti-CCP2 IgG, IgM, IgA; RF IgM, IgA; anti-CarP IgG, IgM, IgA; range 1-8) and per family (anti-CCP2 and anti-CarP range 1-3; RF range 1–2). Second, the number of IgG anti-modified peptide responses, both in total (anti-CCP2 IgG, anti-CarP IgG, anti-citrullinated-vimentin 59-74 IgG, anti-citrullinated-fibrinogen β 36-52 IgG, and α 27-43 IgG, anti-citrullinated-enolase 5-20 IgG, anti-acetylated-lysine IgG, anti-acetylated-ornithine IgG; range 1–8) and per modification (citrullinated peptides range 1–4; acetylated peptides range 1–2). Differences between categories were calculated using analysis of variance (ANOVA) for continuous outcomes (DAS and ΔDAS 0–4 months), adjusted for age, gender, and smoking (ever/never), and baseline body mass index and Health Assessment Questionnaire (HAQ) score, which were independent predictors of early remission in the IMPROVED study [5]. Binary logistic regression was used for categorical outcomes, adjusted as above, with the analyses of initial DFR additionally adjusted for baseline DAS and the analyses of long-term sustained DFR additionally adjusted for baseline DAS and treatment arm. Holmes-Bonferroni methods were used to correct the alpha level for multiple testing, assuming the same number of hypotheses as pairwise comparisons made. All reported *p* values are derived from the analysis models following correction; only *p* values that remained significant after correction for multiple testing are reported in the figures.

## Results

### Antibody positivity at baseline and 8 months

At baseline in the full cohort, 68% (323/472) of patients were anti-CCP2 IgG positive, 70% (330/469) were RF IgM positive, and 39% (162/416) were anti-CarP IgG positive. Within the patients that were positive for at least one of these autoantibodies at baseline or at 1 year (n = 399), we (re)measured anti-CCP2, RF, and anti-CarP isotypes and anti-citrullinated and anti-acetylated peptide antibodies in baseline serum and in 8-month serum. Since we selected patients based on baseline seropositivity of anti-CCP2 IgG, RF IgM, or anti-CarP IgG, the high rates of positivity for these antibodies at baseline and 8 months are to be expected (Table 1). The lower rates of antibody positivity at 8 months compared to baseline are largely due to seroconversion from positive to negative in this time period.

**Table 1:** Baseline characteristics and antibody positivity

	Baseline (*N* = 356)	8 months (*N* = 209)
RA (2010 criteria), *n* (%)	356 (100%)	-
Female sex, *n* (%)	243 (68%)	-
Age, mean years (SD)	51.2 (13.2)	-
Symptom duration (weeks), median (IQR)	18 (9-35)^a^	-
Ever smokers	165 (47%)^a^	-
DAS, mean ± SD	3.3 (0.9)	-
Anti-CCP2 IgG, *n* (%)	292 (82%)	168 (80%)
Anti-CCP2 IgM, *n* (%)	146 (41%)	62 (30%)
Anti-CCP2 IgA, *n* (%)	150 (42%)	58 (28%)
RF IgM, *n* (%)	267 (75%)	121 (58%)
RF IgA, *n* (%)	212 (60%)^a^	84 (40%)^a^
Anti-CarP IgG, *n* (%)	175 (49%)	64 (31%)
Anti-CarP IgM, *n* (%)	141 (40%)	35 (17%)
Anti-CarP IgA, *n* (%)	109 (32%)^a^	23 (11%)
Anti-cetyl-Lysine IgG, *n* (%)	130 (37%)	67 (32%)
Anti-Acetyl-Ornithine IgG, *n* (%)	252 (71%)	132 (63%)
Anti-Cit-Vim IgG, *n* (%)	208 (58%)	100 (48%)
Anti-Cit-Fib α IgG, *n* (%)	101 (28%)	29 (14%)
Anti-Cit-Fib β IgG, *n* (%)	213 (60%)	105 (50%)
Anti-Cit-Eno IgG, *n* (%)	115 (32%)	58 (28%)
Number of isotypes, median (IQR)	4 (2–6)^a^	3 (1–4)^a^
Number of AMPAs, median (IQR)	4 (2–-6)	4 (2–5)

*Vim* vimentin, *Fib* fibrinogen, *Eno* enolase, *IQR* interquartile range, *Lys* lysine, *Orn* ornithine, *SD* standard deviation

^a^Some missing values. See Fig. 1 for number of data available on individual antibody measurements. Available data for symptom duration and smoking, *n* = 355; for anti-CarP IgA, *n* = 344 at baseline; for number of isotypes *n* = 343 at baseline; *n* = 208 at 8 months

### Initial change in DAS

**Fig. 2:**
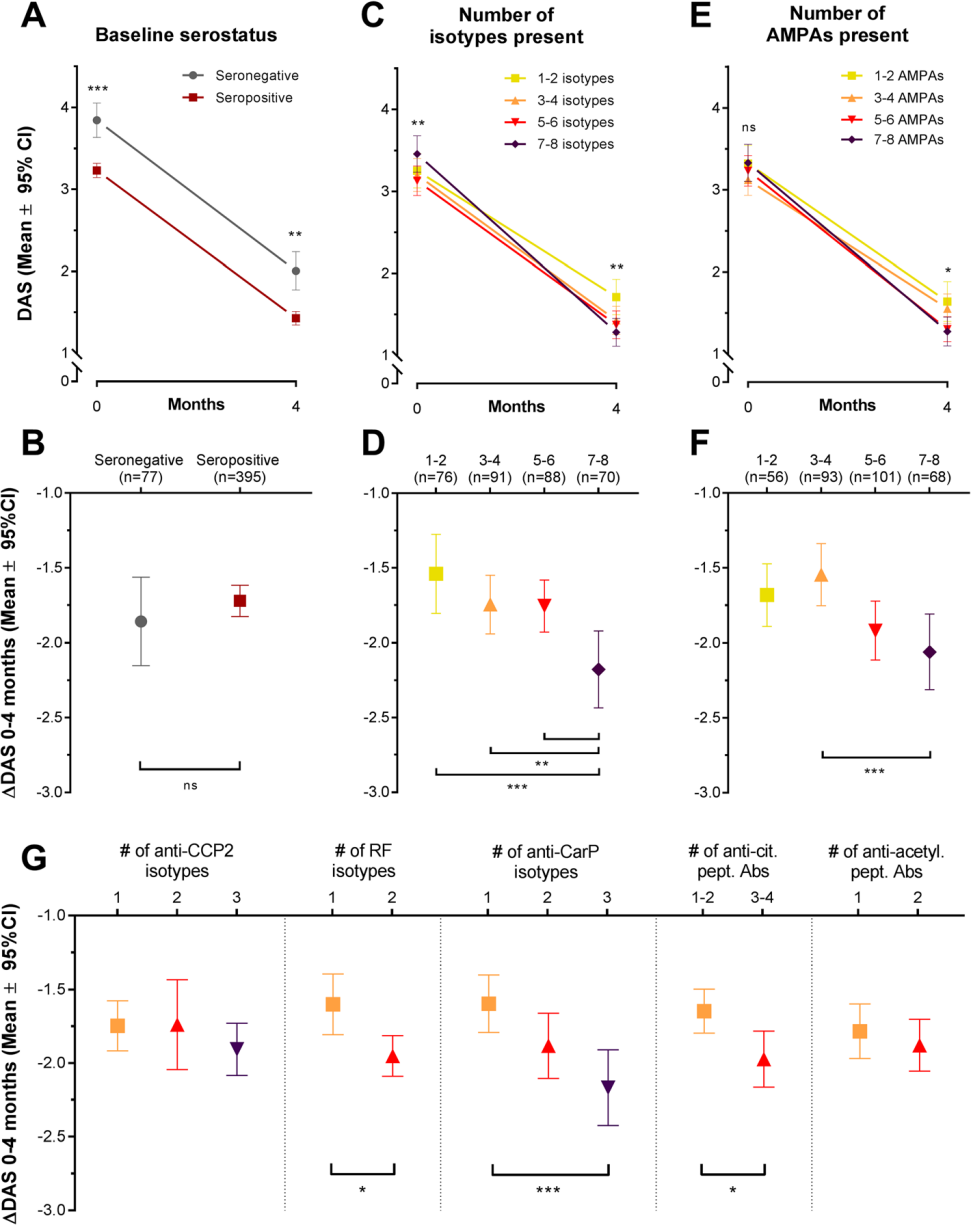
Disease activity score (DAS) (mean +/- 95% confidence intervals) over 4 months of treatment and mean initial change in DAS from baseline to 4 months (ΔDAS 0–4 months), separated by baseline serological status and breadth of autoantibody response. **a**, **b** DAS over time and ΔDAS 0–4 months separated by baseline autoantibody seropositivity based on anti-citrullinated protein 2 (anti-CCP2) IgG, rheumatoid factor (RF) IgM, or anti-carbamylated protein (anti-CarP) IgG positivity. Based on availability of antibody data, the total number of patients included in **a** and **b** is 472. **c**, **d** Within baseline seropositive patients, DAS over time and ΔDAS 0–4 months separated by the total number of isotypes present (anti-CCP2 IgG, IgM, IgA; RF IgM, IgA; and anti-CarP IgG, IgM, IgA). Due to the technical success rate of isotype measurements, and some seropositive patients testing negative upon re-measurement (see Additional file 1: Figure S1), the total number of patients included in **c** and **d** is 325. **e**, **f** Within patients seropositive at baseline, DAS over time and ΔDAS 0–4 months separated by the total number of anti-modified peptide antibodies (AMPAs) present (anti-CCP2 IgG, anti-CarP IgG, anti-citrullinated-vimentin 59-74 IgG, anti-citrullinated-fibrinogen β 36-52 IgG and α 27-43 IgG, anti-citrullinated-enolase 5-20 IgG, anti-acetylated-lysine IgG, and anti-acetylated-ornithine IgG). Thirty-eight patients were RF IgM positive but had no AMPAs (not shown). Due to the technical success rate of isotype measurements and some anti-CCP2 IgG and anti-CarP IgG positive patients testing negative upon re-measurement (see Additional file 1: Figure S1), the total number of patients included in **e** and **f** is 318. **g** Within baseline seropositive patients, ΔDAS 0–4 months separated by the number of isotypes present per antibody family and for the number of antibodies present to citrullinated or acetylated peptides. Reported *p* values are adjusted for multiple testing using Holmes-Bonferroni methods. ns, not significant (*p* ≥ 0.05); *p < 0.05; **p < 0.01, ***p < 0.001. Anti-cit. pept. Abs, anti-citrullinated peptide antibodies; Anti-acetyl. pept. Abs, anti-acetylated peptide antibodies

We first analysed the association between the patients’ baseline autoantibody profiles and initial treatment response. As shown in Fig. 2a, seropositive patients (defined by the presence of anti-CCP2 IgG and/or RF IgM and/or anti-CarP IgG in the original baseline measurement) had a lower DAS at baseline than triple-negative patients. This was most likely due to the ACR/EULAR2010 RA criteria selection we used; seropositive patients require fewer other clinical items to fulfil the criteria and thus have a lower DAS at baseline than seronegative patients. Notably, despite these differences in absolute DAS, the initial change in DAS from baseline to 4 months was equal between seropositive and seronegative patients (Fig. 2b and Additional file 1: Figure S3A), also after correction for relevant covariates.

Strikingly, within the seropositive patients, initial DAS response in patients with many isotypes was more pronounced than in those with few isotypes (ΔDAS 0–4 months of 7–8 isotypes vs 1–2, 3–4, and 5–6 isotypes, respectively: -2.2 vs -1.5 (*p* < 0.001), -1.7 (*p* = 0.003), and -1.8 (*p* = 0.001)) (Fig. 2c, d and Additional file 1: Figure S3B). This pattern remained when analysing the number of isotypes present separately for each antibody family: those with more isotypes had better initial DAS response than those with fewer isotypes, and were statistically significant (after correction for multiple testing) for the RF and anti-CarP families (Fig. 2g).

There was the same dose-dependent association between breadth of the autoantibody profile and DAS decline when analysing the overall number of AMPAs present. Initial DAS response in seropositive patients with many AMPAs was better than in those with few AMPAs, although this was not always statistically significant after correction for multiple testing: ΔDAS 0–4 months of 7–8 AMPAs vs 1–2, 3–4, and 5–6 AMPAs, respectively: -2.1 vs -1.7 (*p* = 0.016), -1.5 (*p* < 0.001), and -1.9 (*p* =0.22) (Fig. 2e, f and Additional file 1: Figure S3C). This pattern was also present when analysing the number of antibodies present per post-translational modification, and was significant for citrullinated peptides: ΔDAS 0–4 months of 3–4 vs 1–2 citrullinated peptides was -2.0 vs -1.6 (*p* = 0.01) (Fig. 2g). No single isotype or antibody was disproportionally associated with a better initial DAS response (Additional file 1: Figure S4A).

### Initial successful drug discontinuation

To investigate whether the autoantibody profile at baseline or at the moment of tapering was also relevant for more long-term treatment outcomes, we next examined whether the autoantibody response is associated with ability to discontinue medication and remain in remission for one year after reaching early remission (initial DFR), independently of factors also associated with this outcome (see “Statistical analysis”). In line with previous findings [2], patients with RA who were positive for anti-CCP2 IgG and/or RF IgM were less likely than their negative counterparts to reach initial DFR, although this difference was not significant: 17% of anti-CCP2 IgG positive versus 20% of negative patients (*p* = 0.14) and 16% of RF IgM positive versus 19% of negative patients (*p* = 0.43). Anti-CarP IgG positive patients were also less likely to reach initial DFR than negative patients (11% vs 22%; *p* = 0.03). Since we selected patients on ACR/EULAR2010 RA criteria, thereby enriching for seropositivity, the differences between anti-CCP2 IgG, RF IgM, and anti-CarP positive and negative patients found here were less pronounced than previously reported in the entire IMPROVED study population because in the current study patients negative for one of these antibodies were by definition positive for another [2].

**Fig. 3:**
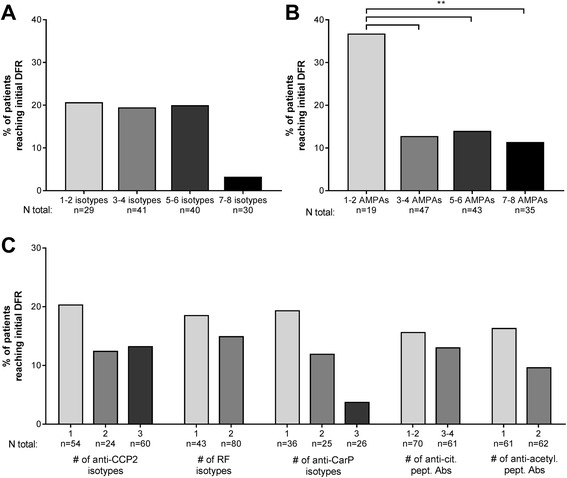
Association between baseline autoantibody profile and initial drug-free remission (DFR) in patients seropositive for anti-citrullinated protein 2 (anti-CCP2) IgG, rheumatoid factor (RF) IgM, or anti-carbamylated protein (anti-CarP) IgG at baseline that had serum available for re-measurement (*n* = 155). Pairwise comparisons between each group were not significant after multiple testing (see text). **a** Percentage of patients with the specified number of isotypes present reaching initial DFR. The composite number of isotypes consists of the positivity count for anti-CCP2 IgG, IgM, IgA; RF IgM, IgA; and anti-CarP IgG, IgM, IgA. Due to the technical success rate of isotype measurements, and some seropositive patients testing negative upon re-measurement (see Additional file 1: Figure S1), the total number of patients included in **a** is 140. **b** Percentage of patients with the specified number of anti-modified protein antibodies (AMPAs) present reaching initial DFR. The composite number of AMPAs consists of the positivity count for anti-CCP2 IgG, anti-CarP IgG, anti-citrullinated-vimentin 59-74 IgG, anti-citrullinated-fibrinogen β 36-52 IgG, α 27-43 IgG, anti-citrullinated-enolase 5-20 IgG, anti-acetylated-lysine IgG, and anti-acetylated-ornithine IgG. Eleven patients were RF IgM positive but had no AMPA antibodies (not shown). **c** Percentage of patients with the specified number of antibodies present reaching initial DFR. Anti-cit. pept. Abs, anti-citrullinated peptide antibodies; Anti-acetyl. pept. Abs, anti-acetylated peptide antibodies. Reported *p* values are adjusted for multiple testing using Holmes-Bonferroni methods. ns, not significant (*p* ≥ 0.05); *p < 0.05; **p < 0.01, ***p < 0.001

Interestingly, while a broad baseline autoantibody response was favourable for initial DAS response, it was unfavourable for the chance of achieving initial DFR (Fig. 3). Within patients seropositive for anti-CCP2 IgG, RF IgM, or anti-CarP IgG at baseline, there was a non-significant trend indicating that patients with more isotypes achieve less initial DFR (1–2, 3–4, and 5–6 isotypes vs 7–8 isotypes, respectively: 21% (*p* = 0.07), 20% (*p* = 0.13), and 20% (*p* = 0.10) vs 3%) (Fig. 3a). Patients with more AMPAs also achieved significantly less initial DFR (1–2 AMPAs vs 3–4, 5–6, and 7–8 AMPAs, respectively: 37% vs 13% (*p* = 0.004), 14% (*p* = 0.007), and 11% (*p* = 0.005) (Fig. 3b).

This trend remained when examining the number of isotypes present separately for each antibody family and the number of antibodies present against citrullinated/acetylated peptides (Fig. 3c). The presence of an anti-CCP2 IgM and/or IgA isotype within patients positive for anti-CCP2 IgG did not decrease the chance of initial DFR, nor did the presence of RF IgA in patients positive for conventional RF IgM (data not shown). Presence of a specific isotype or antibody did not confer increased or decreased chance of reaching initial DFR (Additional file 1: Figure S4B).

**Fig. 4:**
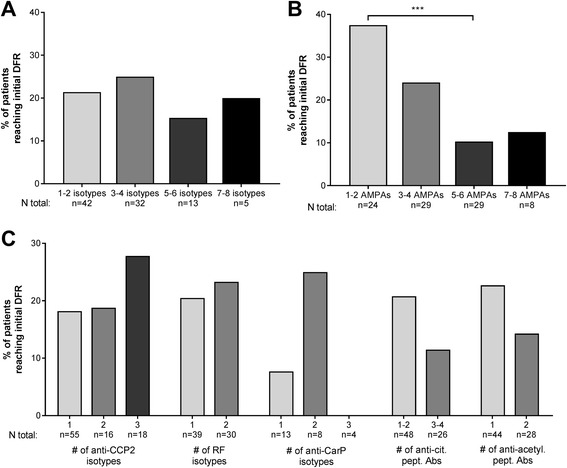
Association between 8-month autoantibody profile and initial drug-free remission (DFR) in patients seropositive for anti-citrullinated protein 2 (anti-CCP2) IgG, rheumatoid factor (RF) IgM, or anti-carbamylated protein (anti-CarP) IgG at baseline, who had serum available for re-measurement at 8 months (*n* = 103). Pairwise comparisons between each group were not significant after multiple testing (see text). **a** Percentage of patients with the specified number of isotypes present reaching initial DFR. The composite number of isotypes consists of the positivity count for anti-CCP2 IgG, IgM, IgA; RF IgM, IgA; and anti-CarP IgG, IgM, IgA. Due to some seropositive patients testing negative upon re-measurement or seroconverting to negative by 8 months, the total number of patients with any isotypes present was 92. **b** Percentage of patients with the specified number of anti-modified protein antibodies (AMPAs) present reaching initial DFR. The composite number of AMPAs consists of the positivity count for anti-CCP2 IgG, anti-CarP IgG, anti-citrullinated-vimentin 59-74 IgG, anti-citrullinated-fibrinogen β 36-52 IgG and α 27-43 IgG, anti-citrullinated-enolase 5-20 IgG, anti-acetylated-lysine IgG, and anti-acetylated-ornithine IgG. Thirteen patients were RF IgM positive but had no AMPA antibodies or had seroconverted to negative by 8 months (not shown). **c** Percentage of patients with the specified number of antibodies present reaching initial DFR. Anti-cit. pept. Abs, anti-citrullinated peptide antibodies; Anti-acetyl. pept. Abs, anti-acetylated peptide antibodies. Reported *p* values are adjusted for multiple testing using Holmes-Bonferroni methods. ns, not significant (*p* ≥ 0.05); *p < 0.05; **p < 0.01, ***p < 0.001

We investigated whether the autoantibody profile at the moment of tapering had a similar association with initial DFR as the baseline profile. Patients received tapered methotrexate at 8 months if they achieved early remission, allowing them to reach initial DFR between 12 months and 2 years. Seropositive patients with more isotypes at 8 months (i.e. the moment of tapering) tended to achieve less initial DFR than those with few isotypes, but this effect was not as clear as observed in relation to the baseline profile (Fig. 4a). Patients with more AMPAs at 8 months achieved slightly less initial DFR (1–2 AMPAs vs 3–4, 5–6, and 7–8 AMPAs, respectively: 38% vs 24% (*p* = 0.025), 10% (*p* = 0.004), and 13% (*p* = 0.023) (Fig. 4b)), but only the comparison of 1–2 AMPAs with 5–6 AMPAs remained significant after correction for multiple testing. When examining the antibody families separately, there was no clear pattern indicating that more isotypes or reactivity against citrullinated/acetylated peptides was associated with less initial DFR (Fig. 4c).

### Long-term sustained DFR

Finally, we wished to determine whether the baseline autoantibody profile was associated with the most favourable long-term outcome of long-term sustained DFR. Fifty-seven percent of patients that had initial DFR also achieved long-term sustained DFR, defined as at least 1 year of DFR lasting until the last follow up, an outcome approximating disease cure. For patients that were not in early remission at 4 months and therefore could not achieve initial DFR, it was still possible to taper medication at a later stage and reach long-term sustained DFR. In the full RA cohort, baseline anti-CCP2 IgG positive patients reached this outcome significantly less often than their negative counterparts (10% vs 26% (*p* < 0.001)); RF IgM and anti-CarP IgG positive patients followed a similar trend (14% vs 19% (*p* = 0.05).

**Fig. 5:**
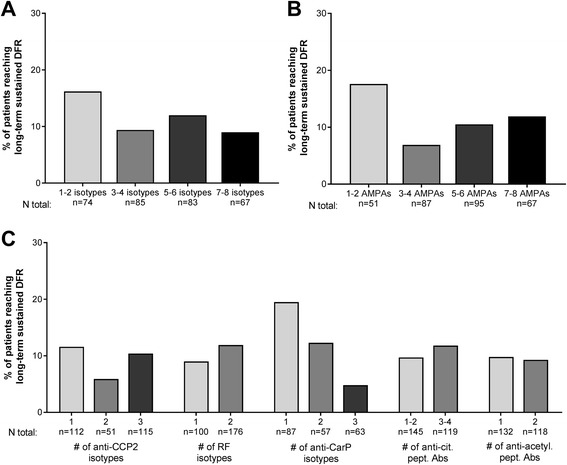
Association of baseline autoantibody profile with long-term sustained drug-free remission (DFR) in patients seropositive for anti-citrullinated protein 2 (anti-CCP2) IgG, rheumatoid factor (RF) IgM, or anti-carbamylated protein (anti-CarP) IgG at baseline (n = 336). Pairwise comparisons between each group were not significant after multiple testing (see text). **a** Percentage of patients with the specified number of isotypes present reaching long-term sustained DFR. The composite number of isotypes consists of the positivity count for anti-CCP2 IgG, IgM, IgA; RF IgM, IgA; and anti-CarP IgG, IgM, IgA. Due to the technical success rate of isotype measurements, and some seropositive patients testing negative upon re-measurement (see Additional file 1: Figure S1), the total number of patients included in **a** is 309. **b** Percentage of patients with the specified number of anti-modified protein antibodies (AMPAs) present reaching long-term sustained DFR. The composite number of AMPAs consists of the positivity count for anti-CCP2 IgG, anti-CarP IgG, anti-citrullinated-vimentin 59-74 IgG, anti-citrullinated-fibrinogen β 36-52 IgG and α 27-43 IgG, anti-citrullinated-enolase 5-20 IgG, anti-acetylated-lysine IgG, and anti-acetylated-ornithine IgG. Thirty-six patients were RF IgM positive but had no AMPA antibodies (not shown). **c** Percentage of patients with the specified number of antibodies present reaching long-term sustained DFR. Anti-cit. pept. Abs, anti-citrullinated peptide antibodies; Anti-acetyl. pept. Abs, anti-acetylated peptide antibodies

In contrast to the previous results on initial DFR (broad baseline autoantibody response: decreased chance of initial DFR), there was no difference in rates of long-term sustained DFR between seropositive patients with many isotypes versus few isotypes, or between patients with many AMPAs versus few AMPAs (Fig. 5a, b). Furthermore, when separately assessing the number of isotypes in a family and the number of antibodies present against citrullinated/acetylated peptides, there were also no differences in long-term sustained DFR rates (Fig. 5c). Only anti-CarP isotypes showed a trend that was no longer present after correction for multiple testing: one isotype versus two isotypes and three isotypes, respectively: 20% vs 12% (*p* = 0.49) and 5% (*p* = 0.02). The presence of an anti-CCP2 IgM and/or IgA isotype within patients positive for anti-CCP2 IgG did not decrease the chance of long-term sustained DFR, nor did the presence of a RF IgA in patients positive for conventional RF IgM (data not shown). Last, positivity to a specific isotype or antibody did not confer increased or decreased chances of reaching long-term sustained DFR (Additional file 1: Figure S4C).

It is conceivable that patients who achieved early remission had different chances of reaching long-term sustained DFR than the full IMPROVED population examined here, and that results would have been different for the patients who achieved early remission. To investigate this, we performed sensitivity analysis of the association between baseline antibody profile and long-term sustained DFR in only the patients who achieved early remission. The results were the same in this group as in the whole cohort (Additional file 1: Figure S5).

## Discussion

The present study explored the link between the humoral autoimmune response and clinical outcomes by investigating whether the breadth of patients with RA with a seropositive autoantibody profile was associated with early and late treatment outcomes. We were able to show, for the first time, that the number of autoantibodies at baseline was independently and dose-dependently associated with a greater decrease in the DAS after 4 months of conventional DMARD therapy. Conversely, a broad autoantibody profile at baseline was associated with a smaller chance of achieving DFR at early stages of attempted drug tapering (initial DFR), but not later in the treatment regimen, where long-term sustained DFR was unrelated to the breadth of the autoimmune response. We also found that reassessing the autoantibody profile at the moment of drug-tapering does not provide additional information about the chance of successfully discontinuing medication to that provided by the baseline profile.

We examined three primary outcomes: initial DAS response, initial DFR, and long-term sustained DFR. Little is known about the relationship between initial DAS response and autoantibody profile in RA. Although some studies suggest that seropositive patients with RA with a high level or large number of autoantibodies have a better response to B cell-depleting therapy [25, 26], this is the first study that shows that the magnitude of seropositivity is favourable for DAS response under conventional synthetic DMARD therapy as well.

As for the second outcome, initial DFR, Figueiredo et al. recently showed that patients with a broad pattern of AMPA response are at high risk of disease relapse in the first year after DMARD tapering [27]. Although the trend we found for initial DFR was significant for the number of AMPAs, our findings do not fully support Figueiredo’s observation that a broad autoantibody profile is unfavourable for DFR because we did not identify a dose-dependent effect. The most likely reason is that we used a different, more stringent outcome (i.e. maintaining DFR for a full year) and that we only measured seropositive patients. As such, we had no patients with zero antibodies at baseline, whereas Figueiredo et al. did have such patients, and the contrast with patients with more antibodies was less striking. We also investigated whether the autoantibody profile at the moment of drug tapering (8 months in the IMPROVED study) instead of at baseline determines the chance of successfully discontinuing medication without disease flare. We found that a broad profile at this moment was not associated with initial DFR. These findings are relevant as they indicate that characterising the autoantibody profile at the moment of tapering does not yield additional information over baseline.

Last, we found that the ability to achieve the third outcome, long-term sustained DFR (at least 1 year of DFR until the last follow up), was independent of the breadth of the baseline autoantibody profile. Instead, baseline seropositivity for anti-CCP2 IgG was the only relevant factor associated with inability to achieve long-term sustained DFR, which is similar to other publications describing presence of anti-CCP2 IgG and RF IgM as a poor prognosticator of long-term drug-free remission [1–5].

**Fig. 6:**
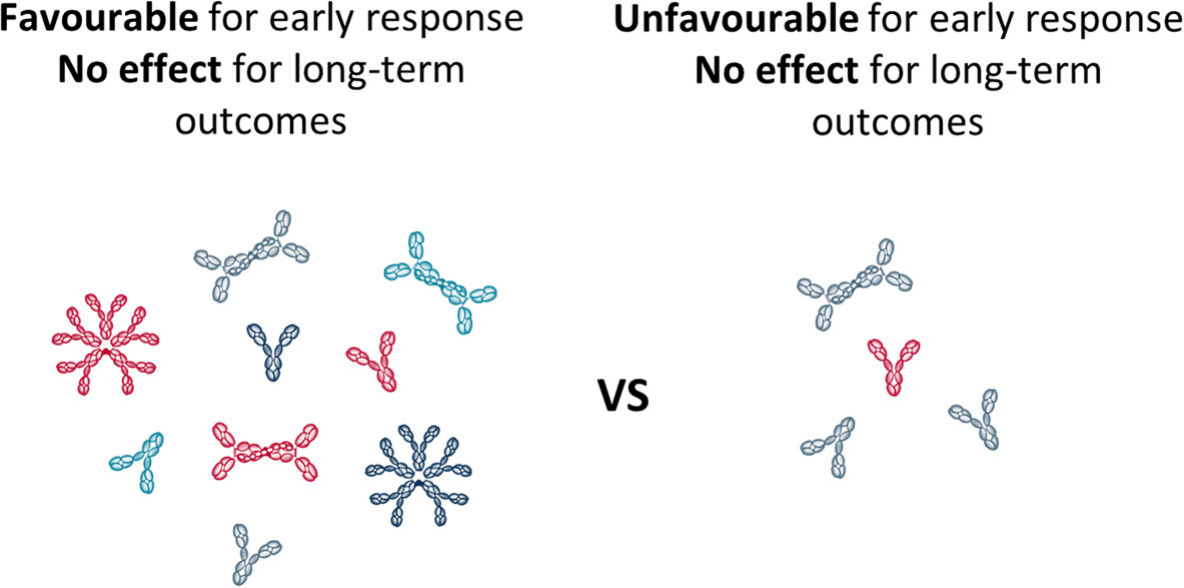
Summary of results. Colours of the antibodies indicate diversity in antigenic targets, and structures indicate diversity in the isotype usage. A broad baseline profile (left) is favourable for early response, but has no association with long-term outcomes like drug-free remission, as compared to a less broad baseline profile (right). The association with the breadth of the baseline profile diminishes with time

Together, these results indicate that the breadth of the autoantibody response in seropositive patients is relevant for early treatment response, somewhat relevant for early attempted drug tapering, but irrelevant for later outcomes (Fig. 6). The presence of multiple antibodies at baseline may indicate an active, ongoing autoimmune response against various post-translationally modified proteins and antigenic targets present in RA, and reflects extensive isotype switching. It is likely that such active immune responses are more susceptible to suppression by methotrexate and prednisone in the initial stages, as evidenced by the stronger early DAS improvement.

The association between the breadth of the autoantibody profile and disease outcomes diminished in magnitude as the outcome investigated became further removed from baseline. This implies that the breadth of baseline autoantibody profile is mostly relevant for short-term outcomes, and has implications for the mechanisms underlying disease chronicity. It has recently been shown that memory B cells expressing anti-citrullinated peptide antibodies (ACPA) persist in the circulation [28, 29], despite conventional DMARD use and remission of synovial disease [30]. These data indicate the persistent presence of a population of auto-reactive B cells that is not affected by therapy. Perhaps the best indicator of this long-lived autoimmunity that accounts for the inability to become drug-free in the long run is presence of this persistent B cell population (the presence of which can be best measured by the anti-CCP IgG test), rather than the recognition of multiple modified antigens or the presence of multiple autoantibody isotypes at baseline. This would explain why anti-CCP2 IgG positivity (firmly established in the literature) but not more antibodies (as in this study) is a poor prognostic factor for DFR.

Another explanation why the breadth of the autoantibody response is only important for early outcomes (i.e. DAS and initial DFR) could be that the autoantibody profile changes during treatment preceding late attempted drug tapering. Indeed, this seemed to be the case as some seroconversion happened between baseline and 8 months. However, considering that the profile at the moment of tapering did not yield more information than the baseline profile for the outcome initial DFR, it does not appear very likely that characterising the profile at even later time points would have yielded more information on later outcomes (i.e. long-term sustained DFR). Furthermore, changes in antibody profile were not the focus of the current investigation.

This study has a few limitations. We chose not to correct for baseline DAS in the case of ΔDAS 0–4 months because doing so may lead to biased results when the explicit outcome of interest is change from baseline [31]. We also performed in-depth serotyping only in patients positive for anti-CCP2 IgG, RF IgM, and/or anti-CarP IgG, so it is possible that we missed some patients who harboured a certain isotype or fine-specificity. However, it has been shown that the occurrence of IgA and IgM anti-CCP2 and responses to citrullinated and acetylated peptides are largely confined to the anti-CCP2 IgG positive subset. No data are available for anti-CarP isotypes, but it seems likely that our broad definition of seropositivity would have captured most anti-CarP isotypes as well, especially since anti-CarP is known to co-occur with anti-CCP2 IgG or RF IgM [32].

Strengths of the current study include that, to the best of our knowledge, it is the broadest autoantibody profile investigation in RA to date (eight isotypes and six fine specificities within four autoantibody systems), in a cohort with an exceptionally long follow up. Furthermore, it is the first study that investigates the relationship between the number of autoantibodies and early response to conventional DMARD therapy. The associations we identified cannot be explained by differences in treatment or in demographic characteristics, as we adjusted all analyses for these. Last, we characterized the antibody profile both at baseline and at the moment of attempted drug-tapering, something that, to our knowledge, has not been investigated thus far.

## Conclusions

This large study shows that seropositive patients with RA with a broader autoantibody profile at baseline initially respond better to treatment and have a slightly worse chance of achieving DFR at early stages, but that the magnitude of seropositivity does not affect the ability to taper off medication and remain in remission later in disease. In early stages of disease, a broad autoantibody profile may reflect active humoral immunity, which could make the underlying disease processes initially more suppressible by medication. The importance of the baseline autoantibody profile for treatment outcomes diminishes over time.

## Additional file

Additional file 1: **Figure S1.** Agreement between previously determined antibody status and remeasurement by ELISA. **Figure S2.** Overlap of isotypes and antibodies at baseline. **Figure S3.** DAS over first year of treatment. **Figure S4.** Initial change in DAS and DFR outcomes within patients positive for individual antibodies. **Figure S5.** Association between baseline autoantibody profile and long-term sustained drug-free remission within patients that reached early remission and had outcome data available. (DOCX 1605 kb)

## Abbreviations

ACR: American College of Rheumatology
AMPA: Anti-modified protein antibodies
ANOVA: Aanalysis of variance
anti-CarP: Anti-carbamylated protein
anti-CCP2: Anti-citrullinated protein 2
DAS: Disease activity score
DFR: Drug-free remission
DMARD: Disease-modifying anti-rheumatic drug
ELISA: Enzyme-linked immunosorbent assay
EULAR: European League Against Rheumatism
HAQ: Health Assessment Questionnaire
IMPROVED: Induction therapy with Methotrexate and Prednisone in Rheumatoid Or Very Early arthritic Disease
MTX: Methotrexate
RA: Rheumatoid arthritis
RF: Rheumatoid factor
