# Supplementary material for: Baseline autoantibody profile in rheumatoid arthritis is associated with early treatment response but not long-term outcomes
Source: Arthritis Res Ther. 2018 Feb 26;20:33. doi: 10.1186/s13075-018-1520-4 (PMC5828136; doi:10.1186/s13075-018-1520-4)
Supplement: Supplementary file 1 — Figure S1. Agreement between previously determined antibody status and remeasurement by ELISA. Figure S2. Overlap of isotypes and antibodies at baseline. Figure S3. DAS over first year of treatment. Figure S4. Initial change in DAS and DFR outcomes within patients positive for individual antibodies. Figure S5. Association between baseline autoantibody profile and long-term sustained drug-free remission within patients that reached early remission and had outcome data available. (DOCX 1605 kb) [file 13075_2018_1520_MOESM1_ESM.docx]

Additional file 1

| **Figure S1:** Agreement between previously determined antibody status and remeasurement by ELISA (n=356).   1. Proportional Venn diagrams displaying agreement between positivity in the previously determined antibody measurement (for anti-CCP2 IgG and RF IgM, by commercial testing; for anti-CarP, in-house ELISA) and the remeasurement (all by in-house ELISA). All samples positive for any antibody were remeasured for the presence of all antibodies. According to Cohen’s κ, there was moderate to good agreement between measurements: anti-CCP2 IgG κ=0.82 (p<0.001); RF IgM κ=0.44 (p<0.001); anti-CarP IgG κ=0.75 (p<0.001). The number of patients included in each Venn diagram may be less than 356 due to missing values in the previously determined measurement. 2. Dotplots representing the arbitrary units (aU/mL) of each antibody for the remeasurement by in-house ELISA within the subset of patients that was previously determined to be negative. Levels upon remeasurement are generally low, suggesting that the discrepancy in positivity between previosly determined and remeasurement may be due to cut-off or inter-test variation. The three patients with high levels upon remeasurement in all three ELISAs are not the same patients. |
| --- |
| 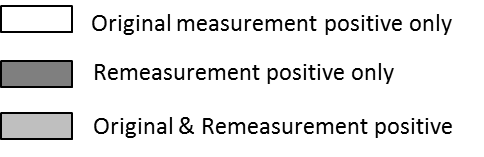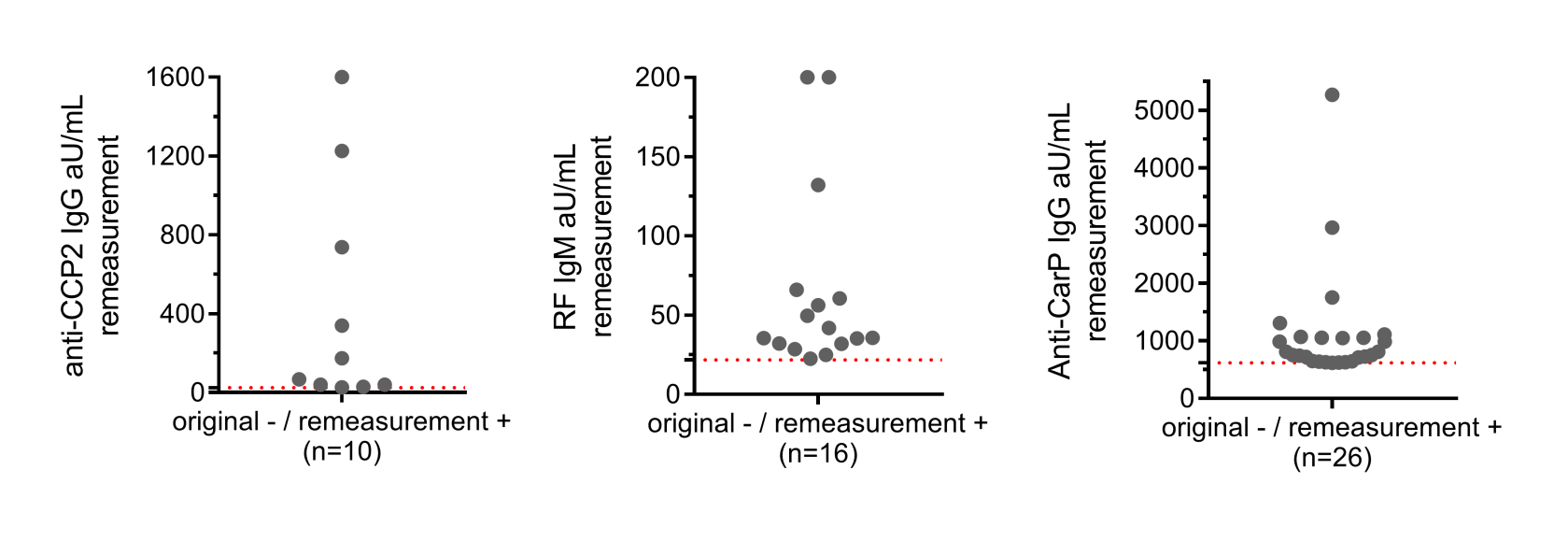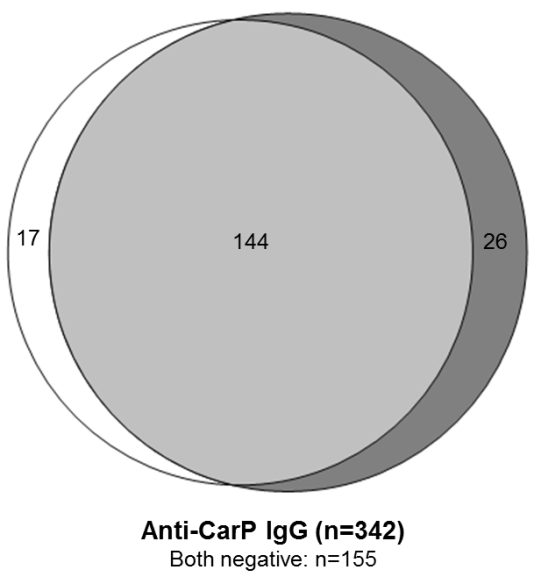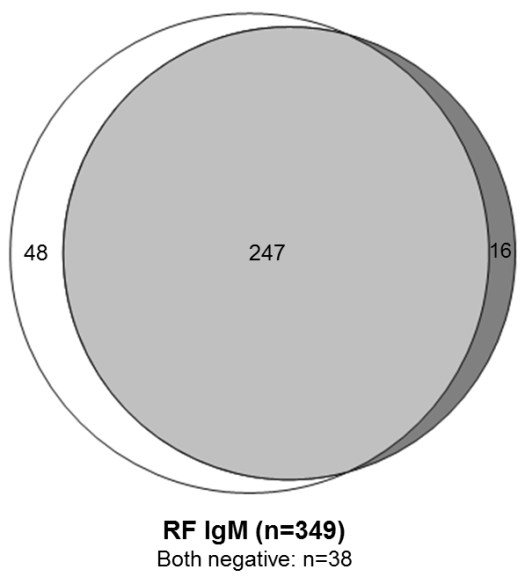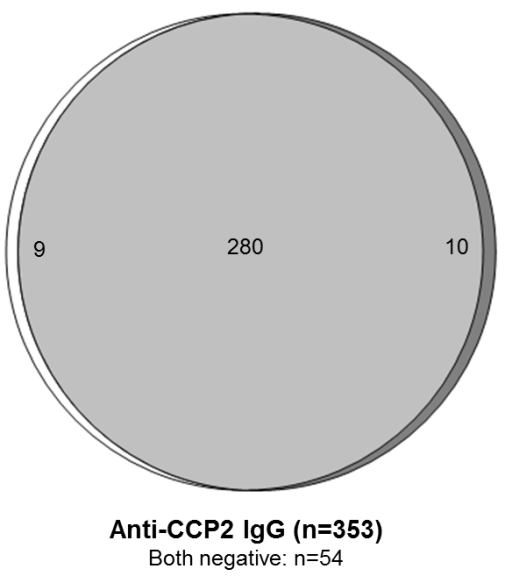 |

| **Figure S2:** Overlap of isotypes and antibodies at baseline upon remeasurement by ELISA. Two- and three-way Venn diagrams are proportional to the number of patients indicated in overlap. There was strong overlap of anti-CCP2 IgG positivity and reactivity to citrullinated peptides. Of 292 anti-CCP2 IgG positive patients, only 8,2% had no reactivity to any citrullinated peptide. Conversely, of 64 anti-CCP2 IgG negative patients, 15,6% harboured reactivity to at least one citrullinated peptide.  Cit = citrullinated. Vim = vimentin; Fib = fibrinogen; Eno = enolase; Lys = lysine; Orn = ornithine. Acetyl = acetylated. Lys = lysine; Orn=ornithine. | |
| --- | --- |
| 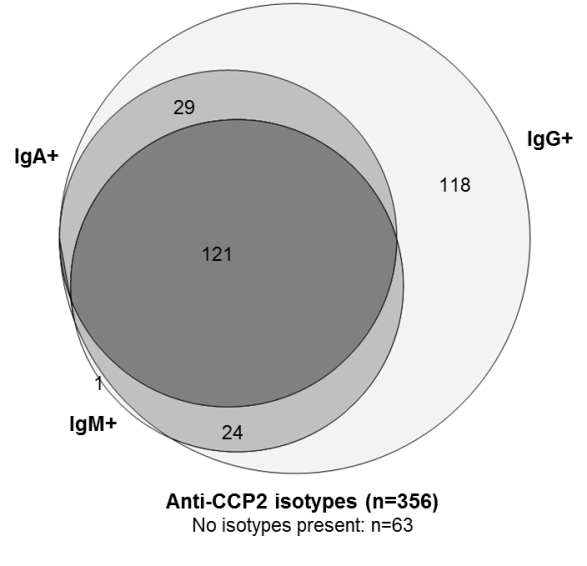 | 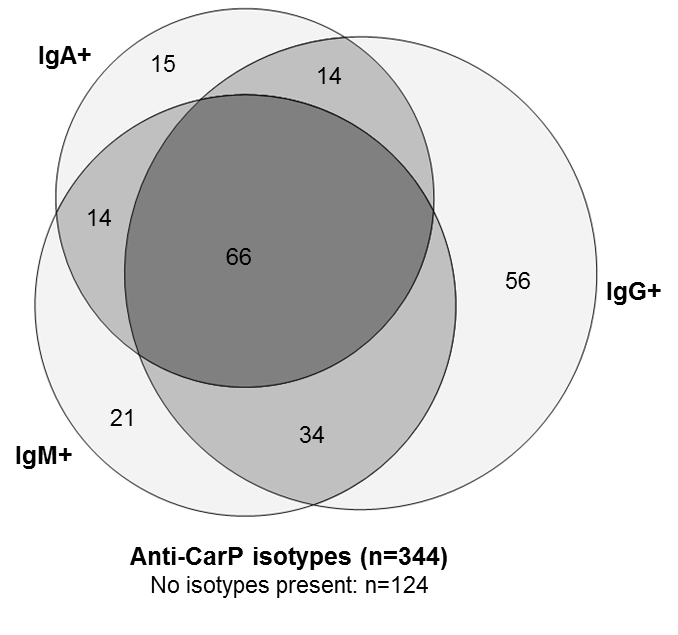 |
| 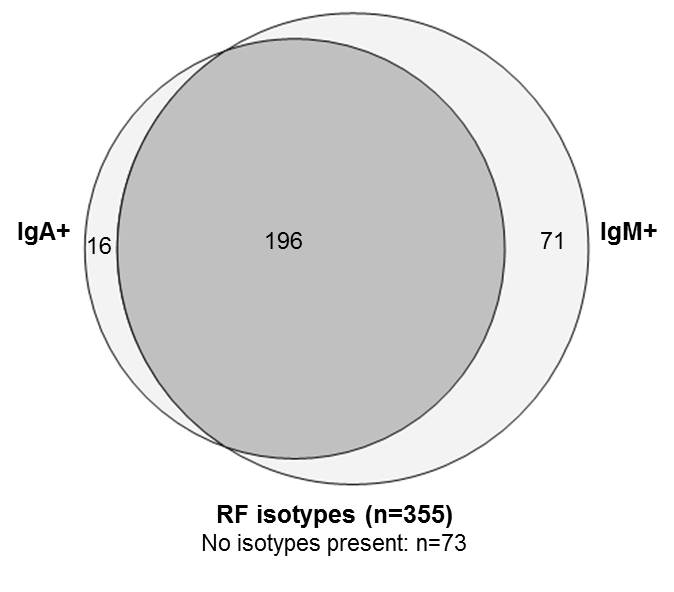 | 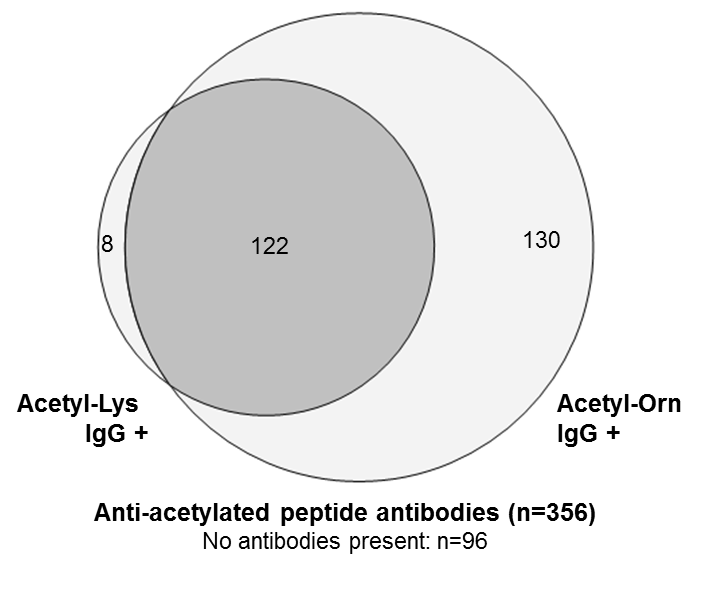 |
| 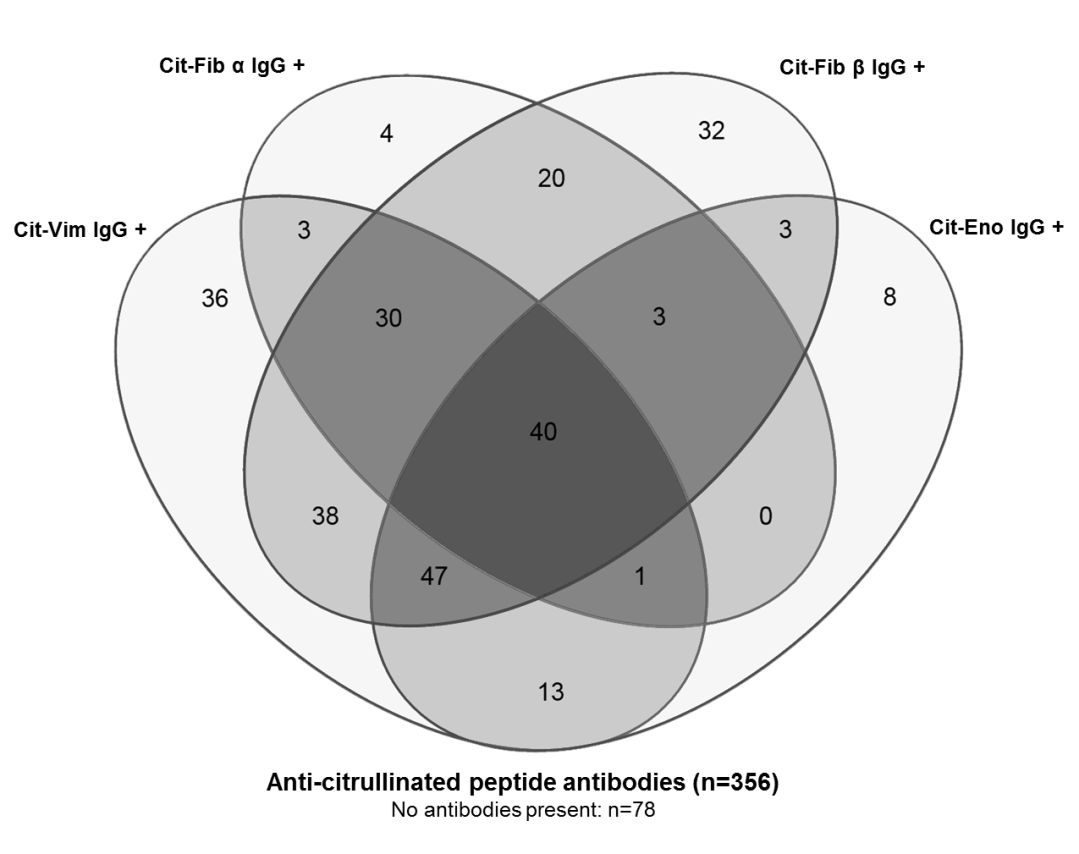 | |

| **Figure S3:** DAS (mean +/- 95% confidence intervals) over first year of treatment (4 month intervals), separated by baseline serological status and breadth of autoantibody response. Adjusted for age, gender, smoking, body mass index, baseline Health Assessment Questionnaire and baseline DAS.  **A)** DAS over time separated for baseline autoantibody seropositivity based on anti-CCP2 IgG, RF IgM, or anti-CarP IgG positivity.  **B)** Within baseline seropositive patients, DAS over time separated for the total number of isotypes present (anti-CCP2 IgG, IgM, IgA; RF IgM, IgA; anti-CarP IgG, IgM, IgA).  **C)** Within baseline seropositive patients, DAS over time separated for the total number of anti-modified peptide antibodies present (anti-CCP2 IgG, anti-CarP IgG, citrullinated-vimentin 59-74, citrullinated-fibrinogen β 36-52 and α 27-43, citrullinated-enolase 5-20, acetylated-lysine, acetylated-ornithine). Thirty-eight patients were RF IgM positive but had no AMPAs (not shown).  Reported P-values are adjusted for multiple testing using Holmes-Bonferroni methods. ns: not significant (p≥0.05); *: p<0.05; **: p<0.01, ***: p<0.001 |
| --- |
|  |

| **Figure S4:** Initial change in DAS and DFR outcomes within patients positive for the indicated individual antibody. No statistical testing was possible as patient groups overlap (see Figure S2).   1. Initial change in DAS (mean +/- 95% confidence intervals) from baseline to 4 months within patients positive for anti-CCP2 IgG, RF IgM, or anti-CarP IgG. 2. Percentage of patients positive for the specified antibody that reached initial DFR. 3. Percentage of patients positive for the specified antibody that reached long-term sustained DFR.   Anti-cit. pept. Abs = anti-citrullinated peptide antibodies; Anti-acetyl. pept. Abs = anti-acetylated peptide antibodies; Vim = vimentin; Fib = fibrinogen; Eno = enolase; Lys = lysine; Orn = ornithine. |
| --- |
|  |

| **Figure S5:** Association of baseline autoantibody profile with long-term sustained drug-free remission within patients that reached early remission and had outcome data available (**A**; n=199) and in only patients seropositive for anti-CCP2 IgG, RF IgM, *or* anti-CarP IgG (**B-D;** n=154). Adjusted for age, gender, smoking, body mass index, baseline Health Assessment Questionnaire and baseline DAS.  **A)** Percentage of anti-CCP2 IgG, RF IgM, or anti-CarP IgG positive and negative patients reaching long-term sustained DFR after early remission.  **B)** Within baseline seropositive patients, percentage of patients with the specified number of isotypes present reaching long-term sustained DFR after early remission. The composite number of isotypes consists of the positivity count for: anti-CCP2 IgG, IgM, IgA; RF IgM, IgA; anti-CarP IgG, IgM, IgA. Due to the technical success rate of isotype measurements and some seropositive patients testing negative upon re-measurement (see Figure S2), the total number of patients included in S6B is 139.  **C)** Within baseline seropositive patients, percentage of patients with the specified number of AMPAs present reaching long-term sustained DFR after early remission. The composite number of AMPAs consists of the positivity count for: anti-CCP2 IgG, anti-CarP IgG, anti-citrullinated-vimentin 59-74 IgG, anti-citrullinated-fibrinogen β 36-52 IgG and α 27-43 IgG, anti-citrullinated-enolase 5-20 IgG, anti-acetylated-lysine IgG, anti-acetylated-ornithine IgG. Eleven patients were RF IgM positive but had no AMPA antibodies (not shown).  **D)** Within baseline seropositive patients, percentage of patients with the specified number of antibodies present reaching long-term sustained DFR after early remission.  Reported P-values are adjusted for multiple testing using Holmes-Bonferroni methods. ns: not significant (p≥0.05); *: p<0.05; **: p<0.01, ***: p<0.001  Anti-cit. pept. Abs = anti-citrullinated peptide antibodies; Anti-acetyl. pept. Abs = anti-acetylated peptide antibodies |
| --- |
| **** |
